# Nature-based activities and mental well-being in adults: a study on perceived health outcomes

**DOI:** 10.3389/fpubh.2025.1611830

**Published:** 2025-06-26

**Authors:** Bilal Okudan, Ozkan Isik, Rifat Yagmur, Canan Bastik Salkim, Laurentiu-Gabriel Talaghir, Teodora Mihaela Iconomescu

**Affiliations:** ^1^Independent Researcher, Ankara, Türkiye; ^2^Faculty of Sport Sciences, Balikesir University, Balikesir, Türkiye; ^3^Directorate of Sports Sciences Application and Research Center, Balikesir University, Balikesir, Türkiye; ^4^Faculty of Sport Sciences, Afyon Kocatepe University, Afyonkarahisar, Türkiye; ^5^Faculty of Sport Sciences, Bursa Uludag University, Bursa, Türkiye; ^6^Faculty of Physical Education and Sport, Dunarea de Jos University of Galati, Galați, Romania

**Keywords:** mental well-being, perceived health outcomes, nature-based physical activity, outdoor recreation, recreational benefit

## Abstract

**Background:**

Nowadays, nature-based sports activities stand out as an essential factor that positively affects individuals’ psychological well-being beyond supporting their physical health. Physical activities in the natural environment can support individuals’ mental well-being by reducing their stress levels, increasing their life satisfaction, and strengthening their psychological resilience. Accordingly, this study aimed to examine the effect of perceived health outcomes from nature-based sport activities on adults’ mental well-being.

**Method:**

A relational screening model was employed with 350 adult participants (183 male, 167 female) engaged in nature-based sports such as skiing, snowboarding, mountaineering, and paragliding in Kocaeli, Türkiye. Participants were selected using a convenience sampling method. Data were collected through a personal information form, the Perceived Health Outcomes of Recreation Scale, and the Mental Well-Being Scale. Both scales demonstrated high reliability in this study (Cronbach’s *α* = 0.93–0.94 for health outcomes; *α* = 0.93 for mental well-being). Normality was verified through skewness and kurtosis values. Data analysis included descriptive statistics, correlation, regression, cluster, and univariate analyses conducted using SPSS 23.

**Results:**

It was determined that there was a positive relationship between perceived health outcomes and mental well-being. In addition, it was observed that perceived health outcomes have a positive effect on mental well-being and predicted mental well-being by approximately 24%. In particular, it was determined that adults who regularly participate in nature-based sports have higher levels of mental well-being in groups with high levels of perceived health outcomes.

**Conclusion:**

It can be stated that regular participation in nature sports creates a positive effect on mental well-being by increasing the level of perceived health outcomes. It can be stated that regular participation in nature sports creates a positive effect on mental well-being by increasing the level of perceived health outcomes. Therefore, promoting accessible and inclusive nature-based sports programs in communities, workplaces, and educational settings is recommended to support public mental and physical health.

## 1 Introduction

The sedentary lifestyle created by urban life increasingly threatens the mental and physical health of individuals (1–3). Intensive urbanization, technological dependencies, and a lifestyle alienated from nature negatively affect the mental and physical well-being of individuals (4, 5). However, the therapeutic effect of time spent in nature strengthens cognitive functions, reduces stress hormones, and supports physical well-being (6–8). This situation necessitates individuals to turn to nature-based activities not only to lead a healthy life but also to increase their psychological resilience (9). One of the conceptual frameworks that helps explain this human tendency is the Biophilia Hypothesis, which suggests that humans have an innate connection with nature and other forms of life. This connection is believed to support cognitive, emotional, and physiological restoration when individuals interact with natural environments (10).

Nature-based activities are becoming increasingly important for mental health as digital technology dependency and virtual engagement in daily life continue to grow. Studies show that interacting with nature lowers individuals’ stress levels, reduces anxiety, improves their general mental health, and increases their life satisfaction (11–13). In addition, Ayhan et al. (14) emphasize that physical activity attitudes also positively influence life satisfaction, suggesting that nature-based activities, which inherently involve physical engagement, may further enhance individuals’ overall well-being. However, the increasing demands of urbanized lifestyles and the growing prevalence of technology dependency have weakened individuals’ connections to nature and diminished the quality of their social relationships (15, 16). At this point, understanding the positive effects of nature-based activities on mental health plays a critical role in strengthening individuals’ stress coping mechanisms. In particular, how participation in recreational nature sports shapes individuals’ psychological well-being has become an increasingly researched topic in recent years (17–19). To strengthen the theoretical foundation, the Biophilia Hypothesis (10), Self-Determination Theory (20), and the Salutogenic Model (21) jointly provide a comprehensive conceptual framework to explain why and how nature-based activities might influence perceived health and mental well-being. These frameworks complement the empirical findings by offering insight into the underlying psychological mechanisms. Previous studies have highlighted that spending time in nature, engaging with natural environments, and participating in outdoor adventure activities can provide significant benefits for stress management, mental health, social connectedness, and overall life satisfaction (22–24). To better understand these psychological benefits, the Self-Determination Theory offers a relevant perspective. This theory emphasizes the role of autonomy, competence, and relatedness as basic psychological needs that, when satisfied through self-directed activities in nature, can promote intrinsic motivation and well-being. Nature-based sports such as skiing, snowboarding, mountaineering, and paragliding are particularly suited to satisfying these needs, offering both challenge and personal achievement (20).

Nature-based activities, including hiking, trekking, mountain biking, cycling, camping, kayaking, canoeing, skiing, snowboarding, paragliding, and mountaineering, vary in terms of intensity and environment. These activities provide opportunities for both low-impact physical engagement and high-adrenaline experiences, supporting mental and physical health through exposure to natural settings, fresh air, and social interaction (25, 26).

Recently, a remarkable increase has been observed in studies on nature-based activities (7, 27–29). Especially with urbanization, the increase in physical and mental health problems caused by modern life has increased individuals’ need for contact with nature. Shanahan et al. (9) stated that activities done in nature can provide physical and mental benefits and thus contribute to healthy aging. Nature-based exercise activities have increased significantly around the world after the 2000s (27, 30, 31). In this context, it is important to consider that individuals’ involvement in activities can positively affect their re-participation intentions. In line with this, Karakullukçu et al. (32) emphasize that increasing involvement in activities enhances individuals’ intentions to re-participate. Many individuals who want to get away from the stress of daily life choose nature therapy methods (33). Nature-based sports in particular stand out as an important factor in supporting individuals’ mental health. Park et al. (34), in their studies examining the physiological and psychological effects of walking in nature, revealed that nature has a relaxing effect compared to the urban environment and leads to positive changes in individuals’ mental processes. In addition, there are various studies in the literature that contact with nature reduces stress, contributes to emotional and cognitive development, increases social skills, and relaxes mentally (35–37). Complementing these perspectives, the Salutogenic Model focuses on how individuals maintain health through the development of personal and social resources. Nature-based activities may act as such resources by enhancing individuals’ sense of coherence and their ability to manage stressors effectively (21). However, the relationship between nature-based activities, mental health, and perceived health outcomes has not been sufficiently explored in the literature. While existing studies generally focus on the relationship between the psychological and physical benefits of nature-based activities, it is seen that there are limited studies that comprehensively examine the effects of these activities on perceived health outcomes and mental well-being depending on the frequency of individuals’ contact with nature. Accordingly, this study aims to make significant academic and practical contributions by examining the relationship between perceived health outcomes from nature-based physical activity and mental well-being. Although the academic literature widely accepts that interacting with nature benefits mental health, there are still important gaps. Specifically, it remains unclear how these benefits are shaped through individuals’ perceived health outcomes.

More specifically, this study seeks to provide both academic and practical contributions by comprehensively examining the relationship between perceived health outcomes derived from nature-based physical activities and mental well-being. Although the positive influence of nature interaction on mental health is well-documented in the academic literature (18, 25, 38), significant gaps remain regarding how these effects are shaped through perceived health outcomes (39, 40). By focusing on this relationship, the study aims to address this empirical gap and provide evidence on how nature-based physical activities contribute to mental well-being through their perceived health benefits. From a practical perspective, the findings are expected to guide physical education and sports professionals, as well as health practitioners, in designing nature-based programs aimed at improving individuals’ physical and mental health, thereby enhancing their overall quality of life. The primary objective of the study is to determine whether perceived health outcomes from nature-based physical activities have a positive impact on adults’ mental well-being.

## 2 Method

### 2.1 Research design and participants

This study was designed as a cross-sectional study with the relational screening model. Relational screening models are a research design used to examine the relationships between two or more variables (41). Cross-sectional studies allow the analysis of existing relationships between variables by collecting data over a certain period. This study was reported in accordance with the STROBE (Strengthening the Reporting of Observational Studies in Epidemiology) guidelines.

In this context, the relationship between the participants’ perceived health outcomes from the nature-based activities and their participation frequency and their mental well-being levels were examined in the study. The study was conducted to evaluate the changes in the health perceptions and mental well-being of adults participating in nature-based sports activities, and a quantitative approach was adopted to understand the relationships between the variables.

The study group of this study consists of adults who participate in nature sports activities in Kocaeli province, Türkiye. The participants comprised adults who regularly participate in nature-based sports such as skiing, snowboarding, mountaineering, and paragliding. Within the scope of the research, participants were reached using the convenience sampling method. This method was preferred due to its practicality and accessibility to active participants in nature-based sports within a specific region. Although this method facilitated data collection, it may limit the generalizability of the findings beyond the current sample. Among the inclusion criteria for the study, adults were required to have participated in at least one nature-based sports activity within the last year, regardless of frequency. The data were collected using a face-to-face questionnaire method, which allowed for more controlled data collection and clarification of any uncertainties during the survey process. The data of a total of 363 adults participating in the study were evaluated, but after the incomplete and incorrectly filled questionnaires were removed, data from 350 participants were included in the analysis. In determining the sample size, the view of Sekaran (42) was taken into account, which states that a sample size of 384 is sufficient with 0.95 confidence when the population exceeds one million. Of the participants, 183 (52.3%) were male and 167 (47.7%) were female. The average age of the research group was 33.96 ± 7.99. When examined in terms of participation frequency, 13 (3.7%) of the adults participating in the study reported participating in nature-based sports once a week, 135 (59.5%) twice, and 70 (36.8%) 3 times or more. This result shows that the participants regularly participate in nature-based sports at a high rate. As a city, Kocaeli has suitable areas for nature-based sports due to its geographical structure, and the Kartepe region in particular is an important center for skiing and snowboarding activities. In addition, activities such as mountaineering and paragliding are also carried out in other parts of Kocaeli.

### 2.2 Data collection tools

These scales were selected because they are widely recognized, validated, and reliable tools for measuring perceived health outcomes and mental well-being. The Perceived Health Outcomes of Recreation Scale has been adapted to the Turkish context and demonstrated high reliability (43), making it suitable for assessing the health benefits of recreational activities. Similarly, the Mental Well-Being Scale is a well-established measure used in various populations to assess general mental well-being, with proven psychometric properties (44, 45). Therefore, these tools were deemed appropriate for capturing the constructs relevant to the aims of this study.

#### 2.2.1 Personal Information Form

In this study, a Personal Information Form was used to determine the demographic information of the participants. The form includes variables such as gender, weekly participation frequency, and purpose of participation in activities.

#### 2.2.2 Perceived Health Outcomes of Recreation Scale

The Perceived Health Outcomes of Recreation Scale, developed by Gómez et al. (46) and adapted to Turkish culture by Yerlisu Lapa et al. (43), was used to measure the participants’ perceived health outcomes from nature-based activities. This scale is a 7-point Likert-type scale consisting of 16 items and three sub-dimensions. There are no reverse-scored items in the scale. High scores obtained from the scale indicate high perceived health outcomes. The Cronbach’s alpha reliability coefficients for the sub-dimensions of the Turkish scale range from 0.89 to 0.91. In the current study, Cronbach’s Alpha reliability coefficients were found to be between 0.93 and 0.94. These values indicate that the Perceived Health Outcomes of Recreation are highly reliable (47).

#### 2.2.3 Mental Well-Being Scale

The Mental Well-Being Scale, developed by Tennant et al. (45) and adapted to Turkish by Keldal (44), was used to determine the mental well-being levels of the participants. The Mental Well-Being Scale has a single-factor structure consisting of 14 items and is a 5-point Likert-type. There are no reverse-scored items in the scale. High scores obtained from the scale indicate high mental well-being. The Cronbach’s Alpha reliability coefficient of the Turkish version of the mental well-being scale was determined as 0.92. In the current study, the Cronbach’s Alpha reliability coefficient was found to be 0.93. This value shows that the Mental Well-Being scale is highly reliable for this study (47).

To provide a clearer overview of the measurement tools used in this study, the items, subdimensions, and indicators of the Mental Well-Being Scale and the Perceived Health Outcomes of Recreation Scale were summarized in the following tables (Tables 1, 2).

**Table 1:** Perceived health outcomes of recreation scale – item summary.

No	Item	Subdimension	Cronbach’s α
1	Helps me appreciate my life more.	Psychological experience	0.940
2	Makes me enjoy life more.		
3	Builds my self-confidence.		
4	Increases my self-respect.		
5	Helps me become more aware of who I am.		
6	Is related to other positive aspects of my life.		
7	Increases my life satisfaction.		
8	Reduces my risk of developing diabetes.	Prevention of negative health outcomes	0.932
9	Reduces my risk of gaining weight.		
10	Reduces my risk of having a heart attack.		
11	Reduces my risk of early death.		
12	Reduces my risk of getting sick.		
13	Improves my fitness.	Improved physical health condition	0.935
14	Improves my general health.		
15	Improves my muscle strength.		
16	Improves my physical flexibility.		

**Table 2:** Mental well-being scale – item summary.

No	Item	Cronbach’s *α*
1	I feel optimistic about the future.	0.930
2	I feel useful.	
3	I feel relaxed.	
4	I am interested in other people.	
5	I have energy to spare for different activities.	
6	I deal with problems well.	
7	I think clearly.	
8	I am satisfied with myself.	
9	I feel close to other people.	
10	I feel confident.	
11	I make my own decisions.	
12	I feel loved.	
13	I am interested in new things.	
14	I feel cheerful.	

### 2.3 Ethical approval

This study was ethically approved by the Balıkesir University Health Sciences Non-invasive Research Ethics Committee with the decision numbered E-52859568-050.04-502767. The research was conducted following the guidelines of the revised Declaration of Helsinki.

All participants were informed about the purpose and scope of the study before data collection. Participation was entirely voluntary, and written informed consent was obtained from each participant. No monetary or material incentives were provided for participation. Data were collected anonymously via face-to-face questionnaires, ensuring confidentiality and the ethical use of all information. The collected data will not be shared with third parties and will be used solely for scientific research purposes.

### 2.4 Statistical analysis

The data obtained in the study were analyzed using the IBM SPSS 23 statistical program. Firstly, the normality distribution of the data was evaluated by examining the Skewness and Kurtosis values. As a result of the normality test, it was determined that the Skewness and Kurtosis values obtained were between +2 and −2, and it was determined that the data were suitable for normal distribution (48). Accordingly, parametric tests were used in statistical analyses. In the study, descriptive statistics were given regarding the demographic information and dependent variables of the participants. Descriptive statistics (frequency, percentage, mean, and standard deviation) were used to determine the perceived health outcomes, participation frequency, and mental well-being levels of the participants. Hierarchical and non-hierarchical cluster analyses were used to separate the participants into specific groups and to reveal the patterns in the data set. These analyses contributed to a better understanding of the differences between the research variables by classifying the participants into different profiles. In addition, Univariate Analysis was performed to evaluate the interaction between perceived health outcomes, participation frequency, and mental well-being. Additionally, Pearson Correlation Analysis was used to determine the relationships between variables, and regression analysis was used to determine the effect of independent variables on the dependent variable and the level of prediction. In all analyses, the significance level was accepted as *p* < 0.05.

## 3 Results

In this section, the relationships between participants’ perceived health outcomes from nature-based activities and their mental well-being levels were first examined using correlation and regression analyses. Following this, a cluster analysis was conducted based on the perceived health outcomes to identify participant profiles with differing levels of perceived benefits. Finally, a Univariate ANOVA was performed to examine the interaction effects of gender and the identified health outcome clusters on mental well-being. The results of these analyses are presented below with the related tables and figures.

The demographic characteristics of the participants indicate a relatively balanced distribution in terms of gender, with 52.3% identifying as male and 47.7% as female. An examination of the frequency of weekly participation in nature-based activities reveals that a significant portion of the sample (59.5%) engages in such activities twice per week. Furthermore, 30.8% report participating three or more times weekly, whereas only 5.7% engage once a week. Regarding participation motives, the majority (62.9%) stated that they engage in nature-based sports primarily to support and improve their physical health. Socialization was identified as a motive by 23.7% of the respondents, while 13.4% reported recreational and mental health-related reasons (Table 3).

**Table 3:** Demographic information of the participants.

Demographic variables		*f*	%
Gender	Male	183	52.3
	Female	167	47.7
Weekly participation frequency	Once	13	5.7
	Twice	135	59.5
	3 or more	70	30.8
Purpose of participation in nature-based activities	Socialization	83	23.7
	Physical health	220	62.9
	Mental health	47	13.4

When the correlation analysis results were examined, it was determined that there was a positive, moderately statistically significant relationship between perceived health outcomes from physical activity and mental well-being (*p* < 0.05; Table 4).

**Table 4:** Correlation analysis results for the relationship between perceived health outcomes and mental well-being.

Variables		Perceived health outcomes	Mental wellbeing
Perceived health outcomes	*r*	1	
	*p*		
Mental wellbeing	*r*	0.495^*^	1
	*p*	0.001	

*p < 0.05.

In order to determine the effect of perceived health outcomes from nature-based physical activities on mental well-being, linear regression analysis was used. As a result of the analysis, it was determined that adults’ perceived health outcomes from physical activities predicted their mental well-being levels by approximately 24% (Adj.R^2^ = 0.243). In other words, it was determined that the independent variable positively affected the dependent variable (*p* < 0.05; Table 5).

**Table 5:** Results of regression analysis on the effect of perceived health outcomes on mental well-being.

Independent variables	*β*	*t*	*p*	*F*	Adj.R^2^
(Constant)	2.626	16.426	0.001^*^	113.067	0.243
Perceived health outcomes	0.282	10.633	0.001^*^		
Dependent variable: Mental well-being				Method: Enter	

*p < 0.05.

The distribution means of the clusters regarding the Perceived Health Outcomes level results were given in Table 6.

**Table 6:** Final cluster centers.

Sub-dimensions of perceived health outcomes scale	Cluster 1	Cluster 2	Mean
The realization of psychological experience	6.20	4.32	5.73
Preventing a worse situation	6.53	4.18	5.94
Improved condition	6.74	4.79	6.25
7-point Likert Type			

It was determined that there were 88 (25.1%) participants in the first cluster and 262 (74.9%) participants in the second cluster (Table 7). It was seen that the distances between these cluster centers were 3.581. As a result of the cluster analysis, the participants’ perceived health outcomes were determined in two different groups as low and high.

**Table 7:** Distance between clusters and cluster size.

Cluster	*n*	Cluster size (%)	Distance between clusters	
			Cluster 1	Cluster 2
Low	88	25.1		3.581
High	262	74.9	3.581	
Total	350	100		

When the analysis results were examined, it was determined that there was a statistically significant difference in the mental well-being outcomes levels of the participants according to their perceived health levels from nature-based activities and their gender (*p* < 0.05). On the other hand, it was observed that there was no significant difference in the mental well-being variable regarding the interaction of perceived health outcomes and gender (*p* > 0.05; Table 8).

**Table 8:** Comparison of the mental well-being levels of the perceived health outcomes clusters from nature-based activities according to gender.

Source	Type III sum of square	df	MS	*F*	*p*	*η* ^2^
Corrected model	27.050	3	9.017	30.110	0.000	0.207
Intercept	4437.975	1	4437.975	14820.118	0.000	0.977
Perceived health outcomes cluster	22.948	1	22.948	76.631	0.000	0.181
Gender	1.181	1	1.181	3.943	0.048	0.011
Perceived health outcomes *Gender	1.152	1	1.152	3.849	0.051	0.011
Error	103.612	346	0.299			
Total	6597.862	350				
Adj.Total	130.662	349				

df, Degree of Freedom; MS, Mean Square.

**Figure 1:**
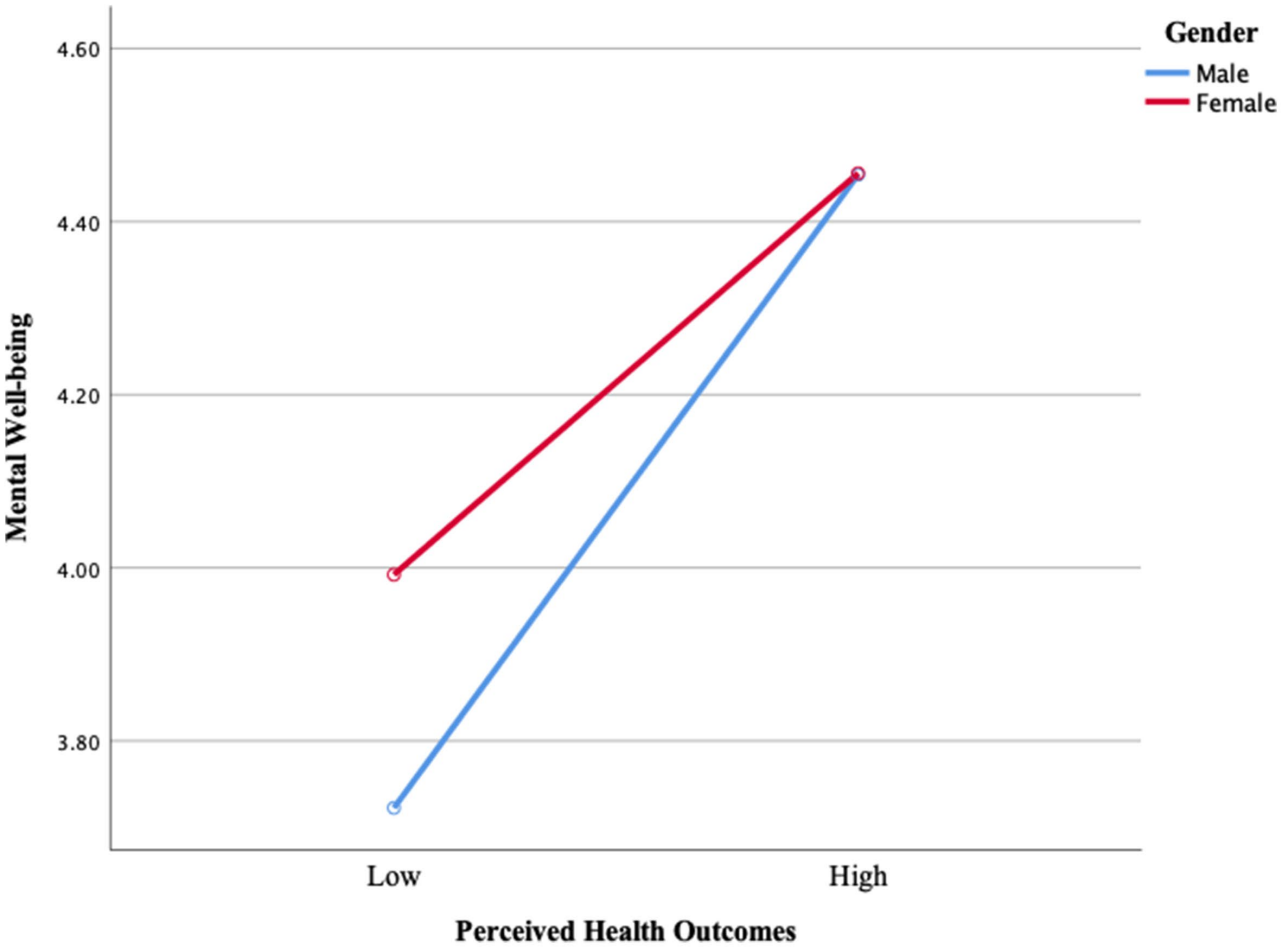
Change in perceived health outcomes clusters and mental well-being according to gender.

Figure 1 shows that mental well-being increases with increasing perceived health outcomes. Men have lower levels of mental well-being compared to women, while they have lower perceived health, and this difference closes as their perceived health increases. Although women have higher initial levels, the increase in perceived health positively affects mental well-being for both genders. This finding suggests that interventions aimed at increasing perceived health, especially for men, may have a more significant effect on strengthening mental well-being.

When the analysis results were examined, it was observed that there was a statistically significant difference in the mental well-being levels of the participants according to their perceived health outcomes from nature-based activities (*p* < 0.05), whereas there was no statistically significant difference in the mental well-being levels according to the frequency of participation (*p* > 0.05). On the other hand, it was determined that there was no significant difference in the mental well-being variable regarding the interaction between the perceived health outcomes and frequency of participation (*p* > 0.05; Table 9).

**Table 9:** Comparison of the mental well-being levels of the perceived health outcomes clusters from nature-based activities according to participation frequency.

Source	Type III sum of square	df	MS	*F*	*p*	*η* ^2^
Corrected model	27.220	5	5.444	18.104	0.000	0.208
Intercept	1996.582	1	1996.582	6639.734	0.000	0.951
Perceived health outcomes cluster	13.269	1	13.269	44.127	0.000	0.114
Participation frequency	1.344	2	0.672	2.235	0.109	0.013
Perceived health outcomes *Gender	0.305	2	0.152	0.507	0.603	0.003
Error	103.442	344	0.301			
Total	6597.862	350				
Adj.Total	130.662	349				

df, Degree of Freedom; MS, Mean Square.

**Figure 2:**
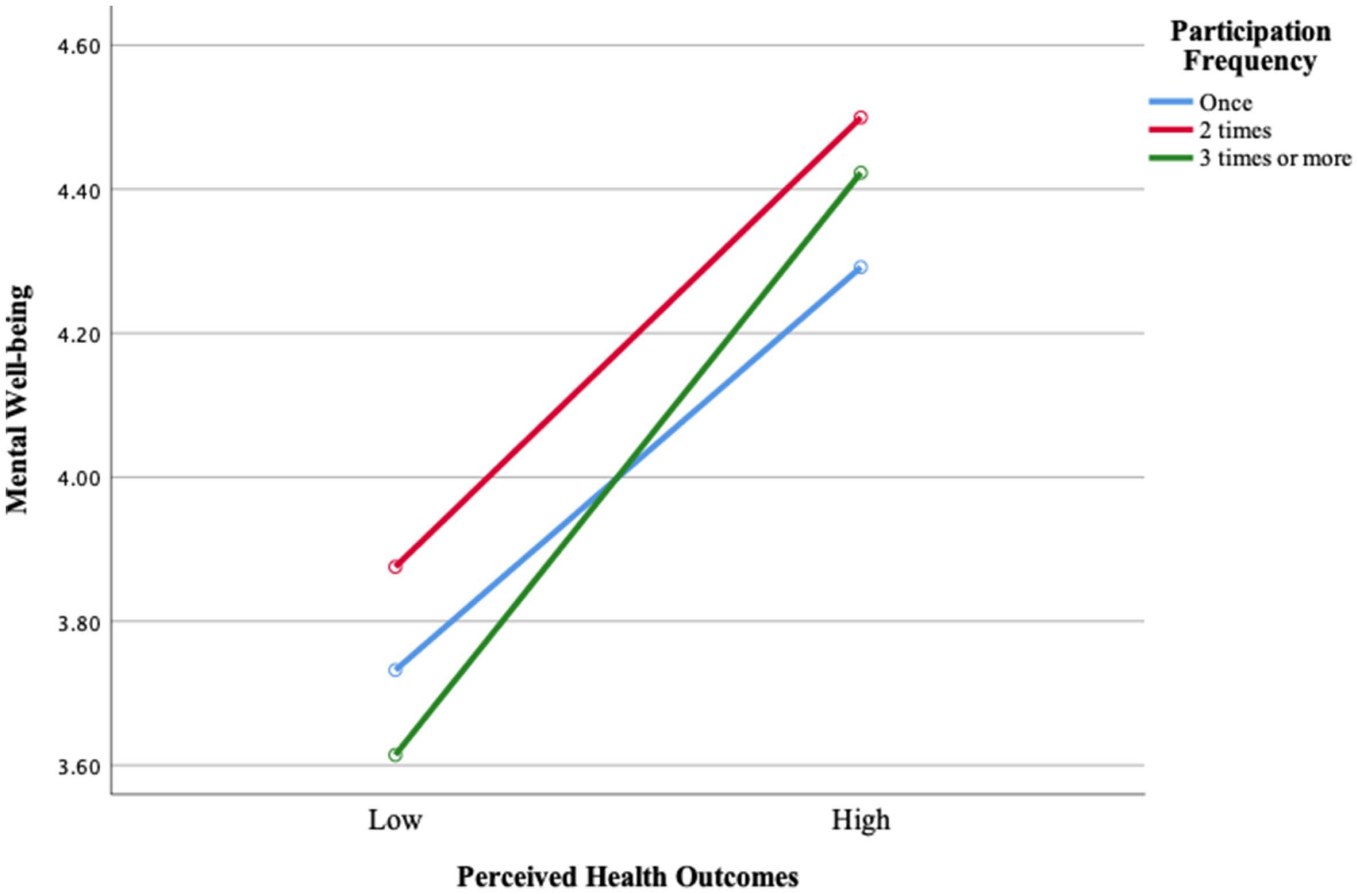
Change in perceived health outcomes clusters and mental well-being according to participation frequency.

When Figure 2 was examined, it was seen that the level of mental well-being increases as the frequency of participation increases. While adults who participate “once” have the lowest level of mental well-being, the level of mental well-being was higher in those who participate “twice,” and the highest level was reached in those who participate “three times or more.” Especially at the level of low perceived health outcomes, the difference between the levels of mental well-being becomes more apparent as the frequency of participation increases. However, at the level of high perceived health outcomes, the levels of mental well-being approach each other as the frequency of participation increases. This finding shows that regular participation is of critical importance in increasing the mental well-being of adults.

## 4 Discussion

The primary purpose of this study was to examine the effects of perceived health outcomes from nature-based physical activities, specifically skiing, snowboarding, mountaineering, and paragliding, on adults’ mental well-being. The findings revealed a moderately significant positive relationship (*r*: 0.495; *p* < 0.001) between perceived health outcomes from nature-based physical activity and mental well-being (Table 4). A review of the literature shows similar findings. For example, Herbert et al. (49) identified a significant relationship between regular physical activity and mental well-being among university students. Similarly, Besikci et al. (50) found that perceived health outcomes from recreational activities were positively associated with psychological resilience and mental well-being during the COVID-19 pandemic. These results suggest that engaging in physical activity in natural environments may help individuals manage stress more effectively and promote mental well-being through positive health perceptions. Another key finding of this study is that perceived health outcomes explained approximately 24% of the variance in mental well-being (Adj.R^2^ = 0.243), with a statistically significant predictive role (*β* = 0.282; *t* = 10.633; *p* < 0.001) (Table 5). This result suggests that individuals who perceive greater health benefits from their participation in nature-based activities may experience enhanced vitality, self-worth, and life satisfaction, all of which contribute to improved mental well-being.

This association can be better understood through several psychological and theoretical frameworks. The Biophilia Hypothesis (10) suggests that humans have an innate connection to nature, which supports cognitive and emotional restoration when engaging with natural environments. Activities such as skiing, snowboarding, mountaineering, and paragliding offer opportunities for such restorative experiences, reducing stress and enhancing psychological well-being. Additionally, the Self-Determination Theory (20) highlights how these activities satisfy basic psychological needs such as autonomy, competence, and relatedness. Participants often choose these activities voluntarily, enhancing their sense of autonomy. Successfully completing physical challenges improves their sense of competence, and participating with others strengthens social connections. Meeting these needs fosters intrinsic motivation and psychological growth, both of which are critical for mental well-being. The Salutogenic Model (21) also emphasizes the role of personal and social resources in maintaining health and managing stress. Perceived health benefits gained through nature-based activities can serve as important resources that strengthen individuals’ sense of coherence—the ability to view life as manageable, comprehensible, and meaningful. Engaging in challenging outdoor activities may help individuals build resilience, develop coping strategies, and adopt a more positive outlook on life. Furthermore, the Ecopsychological Model (51, 52) provides an additional perspective by emphasizing the psychological importance of reconnecting with nature. This model posits that the human mind and the natural world are fundamentally interconnected, and that disconnection from nature can contribute to psychological distress. Participation in nature-based physical activities allows individuals to re-establish this connection, facilitating emotional healing, stress reduction, and improved psychological well-being through immersive interaction with natural environments. Activities such as skiing, snowboarding, mountaineering, and paragliding, which involve direct engagement with natural landscapes, exemplify how such connections can be restored, contributing to holistic health benefits. Participant characteristics also play an important role in interpreting these findings. The sample in this study consisted of adults who voluntarily engaged in nature-based sports, suggesting that they may already value health and well-being. This motivation could amplify the perceived benefits, creating a positive feedback loop between participation, health perceptions, and mental well-being. Additionally, factors such as social interaction, the sense of achievement gained from overcoming physical challenges, and the opportunity to disconnect from urban stressors may all contribute to enhancing mental well-being.

The secondary purpose of this study is to reveal in depth the differences in demographic variables (gender and frequency of participation) on the mental well-being of adults participating in nature-based physical activities. According to the cluster analysis, the health outcomes perceived by adults from nature-based physical activities were divided into two separate classes as low and high (Table 7). According to this classification, it was determined that 25.1% of adults had low and 74.1% had high levels of perceived health outcomes from skiing, snowboarding, mountaineering, and paragliding.

Firstly, it was found that there was a difference between the mental well-being levels of adults with low and high perceived health outcomes (*p* < 0.001) and that the mental well-being of adults also differed according to their gender (*p* < 0.048). However, it was found that the interaction between perceived health outcomes and gender did not differ (Table 8). This result is supported by Figure 1, which shows that the difference between men and women in mental well-being tends to close as perceived health outcomes increase, despite men starting from lower levels. This result may be since men exhibited lower mental well-being compared to women in the case of low health perception and/or the lack of experience of male participants or social expectations. When the literature is examined, some studies show similar results (53–55). For example, Mahalik et al. (56) examined how masculinity norms and socially accepted health behaviors affect men’s health behaviors and stated that men may be more reluctant to express health problems or seek help due to traditional norms, and that this situation may be related to low mental well-being. Addis (57) examined depression in men and how gender norms affect how men recognize, express, and seek help for depression. He found that men often hide their symptoms of depression and have more difficulty coping with emotional difficulties due to social expectations.

Secondly, it was found that there was a difference between the mental well-being levels of adults with low and high perceived health outcomes. On the other hand, the mental well-being of adults did not differ according to their participation frequency, and the interaction between perceived health outcomes and participation frequency was not significant (Table 9). However, Figure 2 shows that the level of mental well-being increases as the frequency of participation increases. Adults who participated once reported the lowest level of mental well-being, while those who participated twice reported higher levels, and the highest level was observed among those who participated three times or more. Especially at the level of low perceived health outcomes, the difference in mental well-being becomes more pronounced as participation frequency increases. However, at the level of high perceived health outcomes, mental well-being levels tend to converge regardless of frequency. These findings suggest that perceived health outcomes from activities such as skiing, snowboarding, mountaineering, and paragliding play a decisive role in mental well-being, while participation frequency does not appear to have a direct effect on this relationship. One possible explanation for this non-significant finding is that the quality of participation may be more influential than the frequency. Individuals who participate less frequently but experience greater personal meaning, emotional engagement, or social connection may benefit more than those who participate frequently without experiencing genuine engagement or satisfaction. This explanation aligns with the Self-Determination Theory (20), which emphasizes that fulfilling basic psychological needs—such as autonomy, competence, and relatedness—fosters intrinsic motivation and psychological well-being. Participants who engage in nature-based activities by their own choice, who experience a sense of achievement, and who connect socially through these activities are more likely to derive psychological benefits, regardless of how often they participate. This result suggests that adults’ motivation for participation, their previous experience with physical activities, and their health perceptions may play a more decisive role in mental well-being. A previous study has reported that individuals with higher levels of physical activity also tend to have higher levels of mental well-being (58). Similarly, other studies have emphasized the positive effects of nature-based activities on mental health (59, 60). However, another study has found a significant impact of weekly participation frequency on mental well-being, which contrasts with the findings of the present study (16). This discrepancy may be explained by individual differences such as personality traits, coping styles, or prior experience with nature-based activities. For example, individuals who prefer solitary or reflective engagement with nature may benefit from fewer but more meaningful experiences, while others may require frequent social interaction to achieve similar psychological benefits.

The non-significant finding in this study suggests that participation frequency may not be a direct determinant of mental well-being but rather may interact with other variables.

### 4.1 Limitations and strengths of the study

This study provides an important contribution in terms of examining the relationship between nature-based physical activities, perceived health outcomes, and mental well-being in detail. However, the study has some limitations. Firstly, the study used a convenience sampling method. Although this method facilitates access to participants and makes the data collection process efficient, it may partially limit the representativeness of the sample for all nature sports participants. Moreover, the study was conducted in a single Türkiye province, which may reflect specific local cultural characteristics, environmental opportunities, and accessibility to nature-based sports such as skiing, snowboarding, mountaineering, and paragliding. These regional factors may have influenced the participants’ experiences, motivations, and health perceptions in ways that are not fully generalizable to other regions or countries. Therefore, future studies should consider sampling participants from different geographic and cultural contexts to better capture diverse experiences and improve the generalizability of the findings. Additionally, there is a potential self-selection bias in the sample, as individuals who voluntarily participate in nature-based sports may already have higher baseline levels of mental well-being, health awareness, or physical activity motivation compared to the general population. This characteristic may have influenced the findings by overrepresenting individuals who are already psychologically or physically advantaged. Future research should consider designs that compare these participants with less active or non-participating populations to better understand the role of prior health status and motivation. Therefore, conducting future studies with a larger sample and different nature-based sports (hiking, camping, cycling, and swimming) participants may increase the generalizability of the findings. Secondly, self-report scales were used in the study. Participants’ responses based on their own perceptions may sometimes lead to social desirability bias or differences in personal assessment. However, such scales are one of the most effective methods that provide valuable and direct information about individuals’ nature-based sports experiences and health perceptions. In further studies, supporting them with hormonal (cortisol, serotonin, dopamine, etc.) changes or qualitative interviews may provide a more in-depth perspective to the studies. Thirdly, the study was conducted with a cross-sectional design. Although cross-sectional studies are a powerful method for understanding the relationships between variables over a period of time, they have some limitations in definitively revealing cause-and-effect relationships. Therefore, it is recommended that future studies evaluate the effects of nature sports over time using longitudinal designs. Fourthly since the data were collected through a single self-report instrument, the possibility of common method bias cannot be fully ruled out. Although the face-to-face administration helped ensure response quality, future studies are encouraged to statistically test for common method bias using techniques such as Harman’s single-factor test. Finally, demographic variables were analyzed at a basic level in the study. However, variables such as age, education level, and socioeconomic status of individuals may affect their motivation to participate in nature sports and their perception of health. In this context, conducting analyses that include more detailed demographic information in future studies will contribute to revealing the differences between individuals in a more comprehensive way.

Although this study has certain limitations, it also has strengths that contribute significantly to the literature in terms of the methodology used, analysis techniques, and the subject matter covered. The strengths of the study increase the scientific reliability of the findings and provide a more comprehensive perspective on the effects of nature-based activities on mental health. First of all, the reliability of the results was increased by using different statistical analyses in the study. The relationships between the variables were tested with Pearson correlation analysis, and the predictive effects of independent variables on the dependent variable were determined with regression analysis. In addition, the participants were classified according to their participation in nature sports with hierarchical and non-hierarchical cluster analyses, and the interactions between perceived health outcomes and mental well-being levels were examined in detail with Univariate Analysis. These methods increase the scientific contribution of the study by enabling the data to be analyzed from different perspectives. Secondly, considering the frequency of adults participating in nature-based activities, individuals who participate in nature sports regularly were compared with those who participate less. This helped determine the effect of the frequency of nature-based activities on the mental well-being of individuals and provided an opportunity to examine this subject, which is addressed in a limited number of cases in the literature, more comprehensively. Thirdly, the scales used in the study were scientifically accepted measurement tools that have been subject to validity and reliability studies. The Perceived Health Outcomes of Recreation Scale and the Mental Well-Being Scale were scales with high reliability values that have been tested on different samples. This ensures that the research is based on a methodologically sound basis. Finally, this study attempts to fill the empirical gap in the literature on the relationships between nature-based activities and health outcomes. Although the contributions of contact with nature to individuals’ mental and physical health are known, the specific effects of participation frequency and perceived health outcomes on mental well-being have not been sufficiently examined. In this context, the findings are expected to guide experts in the field of physical education and sports sciences, nature sports organizers, and health policy makers.

This study provides scientific evidence that nature-based activities can increase both physical and psychological well-being of individuals, indicating that nature sports should be supported by health policies at individual and societal levels.

### 4.2 Recommendations for practitioners

In order to spread the positive effects of nature sports on the mental and physical health of individuals to a wider audience, programs that encourage participation in nature sports should be organized and projects that will facilitate individuals’ access to such activities should be developed. Local governments, sports clubs and private institutions can organize affordable or free activities to ensure that individuals interact more with nature. In addition, integrating nature-based sports into physical education classes in schools and universities will increase individuals’ interest in such activities from a young age and contribute to the development of healthy lifestyle habits. Using nature-based activities not only at the individual level but also as a tool in psychological counseling and therapy processes can be an effective method in managing psychological disorders such as stress, anxiety and depression. In addition, corporate nature sports programs should be developed to support the mental health of employees in the business world and outdoor activities should be encouraged for individuals living in large cities, considering the work-life balance.

## 5 Conclusion

This study examined the effects of perceived health outcomes from nature-based physical activities on the mental well-being of adults. The findings revealed a positive and moderately significant relationship between perceived health outcomes and mental well-being. Regression analysis further indicated that perceived health outcomes accounted for approximately 24% of the variance in mental well-being, demonstrating the significant role of these outcomes in supporting psychological well-being. In addition, the analysis showed significant differences in mental well-being based on perceived health outcomes and gender. Specifically, men reported lower levels of mental well-being compared to women; however, this gap appeared to narrow as perceived health outcomes increased, suggesting that improvements in health perception may play a more critical role in enhancing mental well-being, particularly among men. Although no statistically significant direct effect of participation frequency on mental well-being was identified, graphical analysis indicated that mental well-being levels tended to increase with higher participation frequency. This trend was especially pronounced among individuals with lower perceived health outcomes, highlighting the potential importance of regular engagement in nature-based activities in promoting mental well-being. Overall, these findings contribute to the literature by demonstrating that the psychological benefits of nature-based physical activities such as skiing, snowboarding, mountaineering, and paragliding are closely linked to individuals’ perceived health outcomes rather than the frequency of participation alone. Moreover, the interaction between gender and perceived health outcomes provides additional insight into the differential impact of these activities on mental well-being, offering a valuable contribution to the understanding of gender-specific experiences in the context of nature-based physical activity.

## Data Availability

The raw data supporting the conclusions of this article will be made available by the authors, without undue reservation.
